# Supplementary material for: Designing functional magnetic cloaks for real-world geometries
Source: Sci Adv. 2025 Dec 19;11(51):eaea2468. doi: 10.1126/sciadv.aea2468 (PMC12716416; doi:10.1126/sciadv.aea2468)
Supplement: 20251219-1 [file sciadv.aea2468.v1.pdf]

PHYSICAL SCIENCES

Designing functional magnetic cloaks for real-world geometries

Yusen Guo[1], Alberto Paganini[1]*, Harold S. Ruiz[2]*

Copyright © 2025 The Authors, some rights reserved; exclusive licensee American Association for the Advancement of Science. No claim to original U.S. Government Works. Distributed under a Creative Commons Attribution License 4.0 (CC BY).

Magnetic cloaking enables objects to become undetectable to external magnetic fields while leaving those fields unperturbed. Existing demonstrations rely on idealized cylindrical or spherical shapes, limiting their practical relevance. Here, we introduce a physics-based optimization framework that designs magnetic cloaks for arbitrarily shaped structures by directly solving Maxwell's equations under spatial material constraints. Our method produces continuous, spatially varying permeability profiles that preserve field uniformity around complex geometries, including faceted and multi-lobed configurations. Using material parameters drawn from commercially available superconductors, we demonstrate low-distortion cloaking performance with permeability values within manufacturable ranges. This approach establishes a route to customizable magnetic shields for real-world components and lays the groundwork for future applications in extreme magnetic environments, such as fusion energy systems and precision instrumentation.

INTRODUCTION

In the past two decades, substantial interest has focused on the design of invisibility cloaking devices. Beginning with pioneering works based on methods of transformation optics (*1*), conformal mapping (*2*), and scattering cancelation (*3*), cloaking schemes have expanded from electromagnetic waves to acoustics (*4*), thermal waves (*5*), and even electric (*6*) and magnetic fields (*7*). However, these designs face substantial challenges in practical implementation. Optical and electromagnetic cloaks commonly rely on extreme values of constitutive parameters or highly anisotropic permeabilities (μ) and permittivities (ϵ) of metamaterials (*8*), which undermines their functional feasibility. While conformal mapping or plasmonic scattering cancelation can circumvent some of these challenges, they do so at the cost of achieving only partial invisibility and are often limited to simple geometries or subwavelength-scale objects (*9*).

In the particular case of magnetic cloaking, a superconducting-ferromagnetic heterostructure was first proposed by Sánchez *et al.* (*10*) to address the key fabrication and anisotropy challenges inherent in cloaking electromagnetic waves with metamaterials. Building upon this idea, Gömöry *et al.* (*11*) demonstrated, both analytically and experimentally, that for infinitely long cylinders (practically realized as cylinders whose length greatly exceeds their radius) and with perfectly circular cross sections, two concentric layers made of homogeneous isotropic materials could effectively render an object "invisible" under static magnetic fields. The bilayer cloak consisted of an inner superconducting (SC) shell with permeability $\mu_1 \approx 0$, and an outer soft-ferromagnetic (SFM) shell with permeability $\mu_2 > 1$. Perfect cloaking is achieved in this two-dimensional (2D) configuration when μ_2 satisfies the condition

$$\mu_2 = \frac{R_2^2 + R_1^2}{R_2^2 - R_1^2} \tag{1}$$

where R_1 and R_2 are the outer radii of the SC and SFM layers, respectively (*11*).

This SC-SFM bilayer design was later extended to low-frequency dynamic fields (*12*), with experimental demonstrations confirming ~80% magnetic cloaking effectiveness for ac fields of a few milliTesla and frequencies up to several hundred hertz (*13*). Further advancements led to the realization of more 3D metastructures for static and dynamic fields, naturally extending the 2D analytical concept of cylindrical shells to spherical shells that are likewise symmetrical and coaxial (*14*). Despite these advances, a persistent issue with these designs is their restriction to invariant cylindrical and spherical geometries (*15*), stemming from their fundamental reliance on analytical solutions such as the one at Eq. 1. More recent attempts have been made to extend the bilayer framework to noncircular geometries, although with fundamentally different concepts and scopes. In particular, Zhan *et al.* (*16*, *17*) demonstrated a tailor-made ferromagnetic metasurface that produces a magnetic response that counterbalances the weak diamagnetic signal from a solid brass bar. While effective for a specific, solid object of regular polygonal shape, this approach is distinct from creating a practical magnetic cloak, where an object of any material or shape can be inserted and rendered "invisible" to external magnetic fields. On the other hand, our method aims to eliminate field distortion immediately outside the cloak, aligning with the stricter physical definition of magnetic cloaking as originally proposed in the literature. Therefore, the present work addresses this more general challenge: the design of a magnetic cloak with arbitrarily conforming shape, capable of cloaking itself and any object placed inside it. To this end, we developed an adjoint-based partial differential equation (PDE)–constrained optimization framework under time-dependent magnetic fields (ac), with the full Maxwell's equations serving as constraints. Theoretical foundations for these optimizations including proofs of existence, stability and uniqueness of optimal solutions have been well established for similar problems (*8*, *18*–*24*). This paper presents SC-SFM cloak designs across a range of nontrivial 2D geometries including square and diamond-shaped bodies (see Figs. 1 and 2), multilobed structures (see Fig. 3), and other nonsymmetrical configurations (see fig. S1), demonstrating how magnetic cloaking can be achieved without relying on extreme permeabilities or idealized shapes.

[1]School of Computing and Mathematical Sciences, University of Leicester, Leicester LE1 7RH, UK. [2]School of Engineering, University of Leicester, Leicester LE1 7RH, UK.
*Corresponding author. Email: a.paganini@leicester.ac.uk (A.P.); dr.harold.ruiz@leicester.ac.uk (H.S.R.)

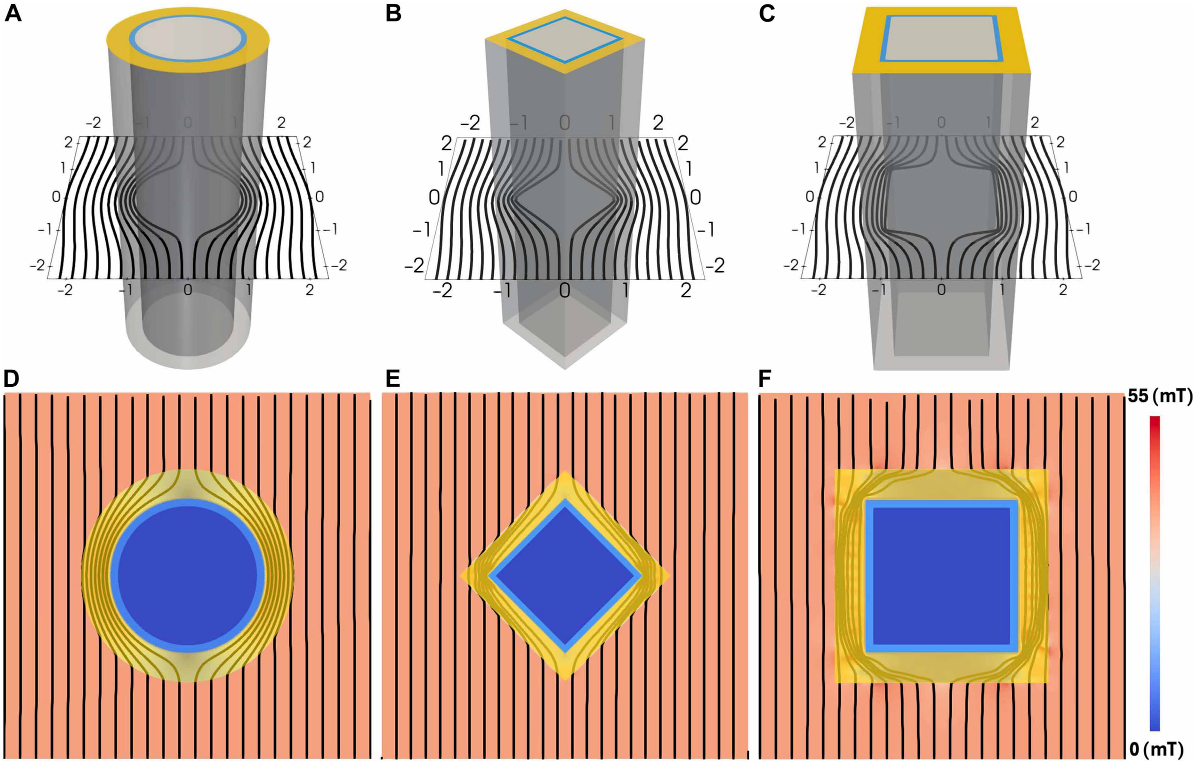

Fig. 1. Magnetic cloaking achieved using bilayer SC-SFM metastructures with different geometries. (**A** to **C**) 3D renderings of cloaking pipes with cross sections of: (A) circular, (B) diamond, and (C) square geometries. The yellow (outer, thicker) shell represents the SFM layer of thickness $R_2 - R_1$, while the blue (inner, thinner) shell denotes the superconducting (SC) domain, with thickness approximately 0.26 mm and inner radius $R_0 = 6.25$ mm, consistent with the experimental conditions reported in (*11*). In that study, $R_2 / R_1 = 1.34$, and the applied magnetic field had amplitude of $B_a = 40$ mT and a linear frequency of 50 Hz. For square cross sections, the "radii" are defined as half the side length, and the geometries are centered in a Cartesian coordinate system with axes normalized to R_1. As shown in the top row, the diamagnetic response of the SC layer alone is disclosed by assigning a relative permeability $\mu_r = 1$(i.e., air-equivalent) to the SFM shell. In contrast, (**D**) to (**F**) shows the optimized magnetic cloaking results for each geometry, obtained by solving the PDE-constrained optimization problem defined in Eqs. 8 to 11. The corresponding optimized permeability distributions are presented in Fig. 2.

RESULTS

To validate our optimization framework and further design functional magnetic cloaking metastructures, we begin with the most classical and well-established configuration: a hollow SC shell coated with a SFM layer in a cylindrical pipe geometry (Fig. 1A), for which a fully analytical solution and experimental validation exist (*11*). We then radically depart from this canonical case by exploring more complex geometries, specifically pipes with diamond-shaped (Fig. 1B) and square-shaped (Fig. 1C) cross sections—chosen based on the orientation of the applied dynamic magnetic field. Although these two geometries may appear similar, they exhibit fundamentally different electromagnetic interactions. In the square-shaped pipe, the external field is either parallel or perpendicular to the flat faces, whereas in the diamond-shaped pipe, the field is oriented diagonally relative to the corners. This distinction demands a different diamagnetic response from the SC layer as can be seen at the top panel of Fig. 1 and, consequently, potentially different material and geometric properties for the surrounding SFM shell to achieve effective cloaking performance as shown in the corresponding bottom panel.

For the bilayer cylindrical model, we initially assume $\mu_{r(SFM)}$ to be a constant function of initially unknown value, with our optimization method rendering to $\mu_{r(SFM)} = 3.48$, meaning a 99.14% accuracy of our model if compared with the analytical solution of $\mu_{r(SFM)} = 3.51$ obtained from Eq. 1, when the outer radius of the SFM shell (R_2) is 1.34 times the exterior radius of the SC layer (R_1). It is well known that no geometry other than the cylinder can achieve perfect or near-perfect magnetic cloaking with a fixed-width ferromagnetic layer when $\mu_{r(SFM)}$ is assumed to be constant. This is why, for tackling the square and diamond-shaped pipes shown in Fig. 1, we removed this restriction in our method and allowed $\mu_{r(SFM)}$ to vary across the SFM domain, leading to cloaking configurations that could be engineered by directly optimizing the $\mu_{r(SFM)}$ profile, as shown in Fig. 2. A nearly perfect solution can be found for the diamond-shaped pipe with a magnetic field distortion of about 0.01% if $\mu_{r(SFM)}$ is allowed to be unbounded and 0.31% for the case of the square shaped/oriented pipe (see also fig. S2). Nonetheless, these optimized permeabilities exhibit sharp peaks and abrupt spatial variations, reflecting material inhomogeneities that could pose manufacturing challenges. To mitigate these challenges, the anisotropy of the magnetic permeability can be computationally modulated by adding a regularization term $\alpha \ \mathcal{R}_\epsilon\left[\mu_{r(SFM)}\right]$ that penalizes excessive spatial variation in $\mu_{r(SFM)}$ by controlling α, such that a quasi-isotropic SFM metastruxture can be found at the expense of increasing the magnetic distortion factor (see Materials and Method). Specifically, we selected $\alpha = 0.5$ for the diamond-shaped cloaks and $\alpha = 0.25$ for the square

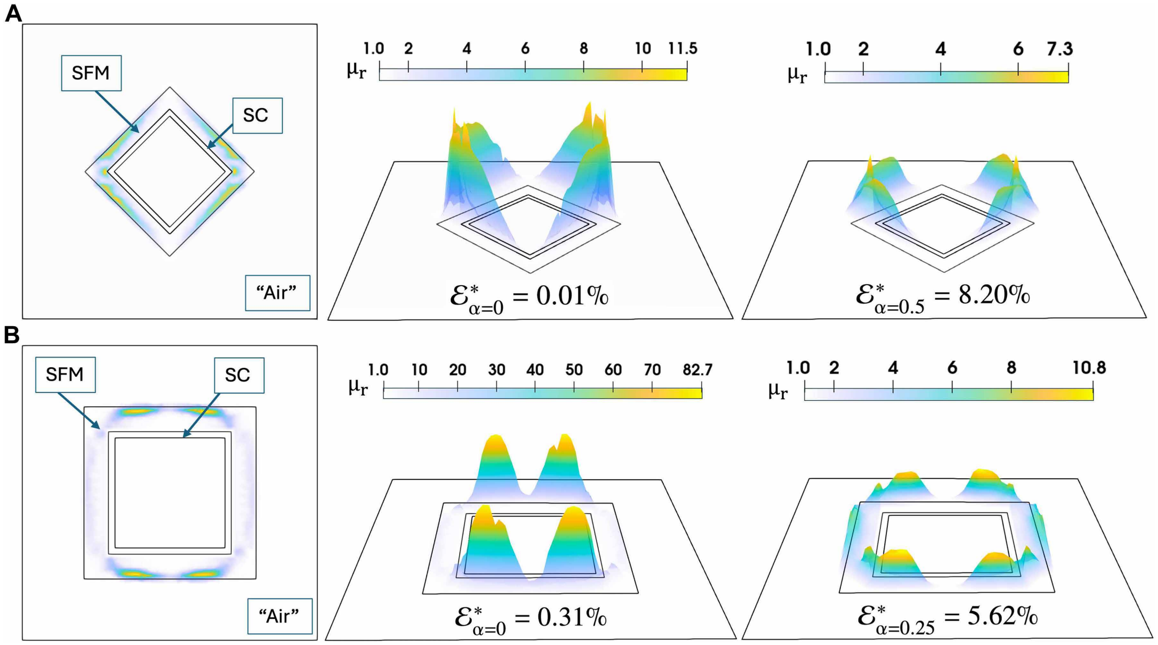

Fig. 2. Optimized permeability profiles for magnetic cloaking in faceted anisotropic geometries. (**A**) Top row: Diamond-shaped cloak. (**B**) Bottom row: Square-shaped cloak. Left column: 2D cross-sectional views of the cloaking pipes showing the optimized $\mu_{r(SFM)}$ distributions obtained without regularization (i.e., $\alpha = 0$ in Eq. 10). Middle column: Corresponding 3D visualizations of the unregularized permeability distributions. Right column: Effects of regularization, which significantly reduce the anisotropy of the SFM layer, lowering the peak values of $\mu_{r(SFM)}$ from 11.5 to 7.3 in the diamond-shaped pipe and from 82.7 to 10.8 in the square-shaped pipe while still maintaining satisfactory cloaking performance. The corresponding normalized distortion metrics remain low at $\mathcal{E}^* = 8.20\,\%$ and $\mathcal{E}^* = 5.62\,\%$ for the diamond and square configurations, respectively, as also reflected in the magnetic field lines shown in the bottom row of Fig. 1.

cloak, as these values provided a suitable trade-off between permeability smoothness and cloaking effectiveness specific to each geometry. The resulting optimized permeabilities are displayed in the right panels of Fig. 2, whose behavior remains unaltered to changes in the intensity of the applied magnetic field up to the values here explored, i.e., 10 mT to 1.5 T (see also fig. S3). Thus, we observe that the introduced regularization functional, $\mathcal{R}_\epsilon\left[\mu_{r(SFM)}\right]$, effectively dampens sharp peaks and oscillations while preserving satisfactory cloaking performance, which is quantified with the normalized distortion metric $\mathcal{E}^* := \mathcal{E}\left[\mathbf{H}, \mu_{r(SFM)}^{optimal}\right] / \mathcal{E}\left[\mathbf{H}, \mu_{r(air)}\right]$, where the denominator represents the distortion in the superconducting-only case [i.e., with] $\mu_{r(SFM)} = \mu_{r(air)}$.

In addition, increasing the thickness of the SFM shell leads to an overall reduction in $\mu_{r(SFM)}$, consistent with the behavior observed in cylindrical cloaks described by the analytical solution in Eq. 1. To provide further insight, we have conducted a benchmark study increasing the thickness of the SFM shell in ~12 and 27% of its original value (table S1), i.e., from 2.21 to 3.26 mm and 4.56 mm in the cylindrical, rectangular, and diamond cloaks, obtaining an average reduction in the local values for the magnetic permeability of ~26 and 38%, respectively. This in the case of the cylindrical configuration with constant permeability []$\mu_{r(SFM)} = 3.48$, which allows for direct comparison with the analytical solution at Eq. 1, that leads to a resulting $\mu_{r(SFM)} = 2.59$ for $d = 3.26$ mm and $\mu_{r(SFM)} = 2.13$ for $d = 4.56$ mm. This inverse relationship holds qualitatively for more complex, noncylindrical geometries as well. In these cases, the optimized $\mu_{r(SFM)}$ becomes spatially varying, but we still observe that thicker SFM layers lead to lower peak values of permeability, with the higher-permeability regions concentrated near the outer boundary.

To further demonstrate the geometric flexibility of our method, Fig. 3 extends the cloaking design to a more intricate cross-sectional shape, inspired by triaxial arrangements of high-voltage conductors such as those shown in Fig. 3A. This geometry, which encompasses scenarios where conductors of different cross sections share the same right-of-way, can be treated as a single, nonsymmetric cloak that someone might say resembles the face of the popular animated character by Disney, Mickey Mouse. Yet, this resemblance is unintended and merely reflects the practical challenge of shielding multiple cables within a single structure. Thus, instead of assuming coaxial cloaks around each conductor, we envision a unified superconducting (SC) and cloaking metastructure (SC-SFM) to address scenarios lacking axial symmetry and involving sharp geometrical transitions—namely, concave and convex facets with distinct corners and edges.

When only the SC layer is used, a highly anisotropic shielding response is observed (Fig. 3B). Strong field deformations emerge near the lateral lobes (dark red regions), while pronounced shielding is seen between them (blue regions), as also visible in the left panel of Fig. 3D. These contrasting behaviors present substantial challenges for achieving effective cloaking, particularly due to the nonuniformity of field intensities around the structure. Nevertheless, as shown in Fig. 3C, a functional cloaking solution is found that restores the background field not only in regions where the magnetic field must be suppressed (near the upper lobes) but also in areas where it must be enhanced, such as beneath and over the central lobe and between the two side lobes, which represent the most

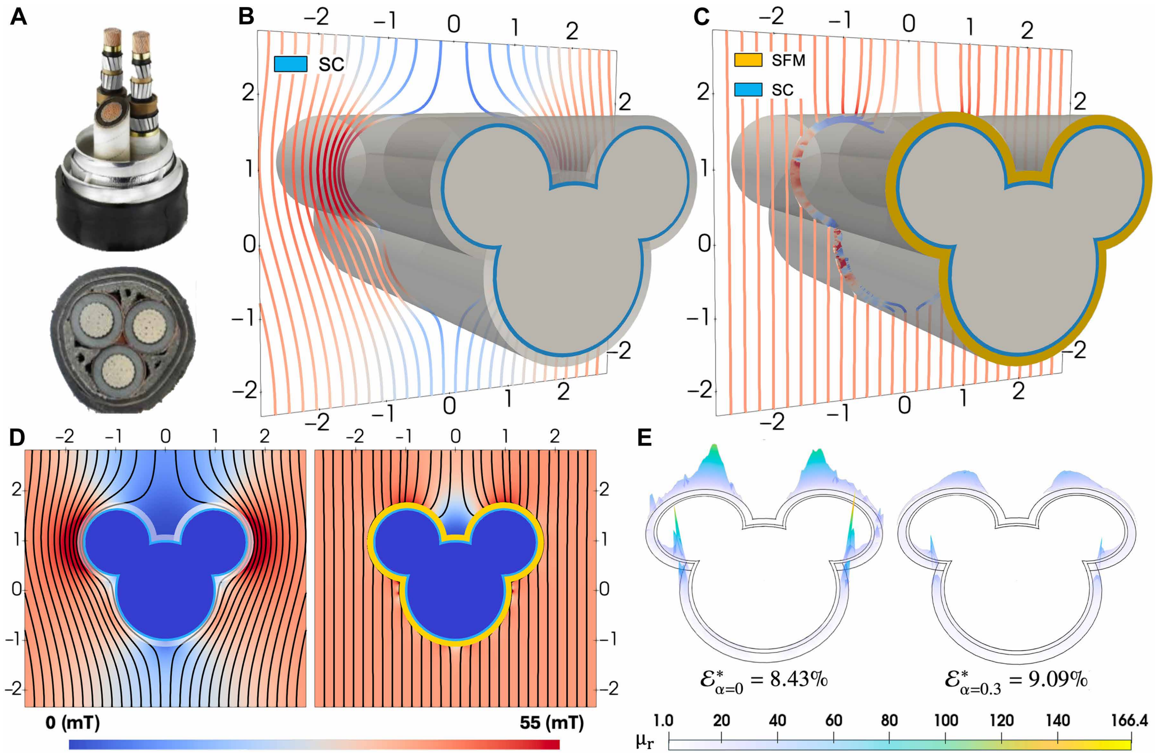

Fig. 3. Magnetic cloaking in a multi-lobed noncircular geometry inspired by realistic cable layouts. (**A**) Real-world examples motivating the modeled cross section: Top: A 23-kV triad-type high-temperature superconducting (HTS) distribution cable (*52*). Bottom: A three-core aluminum-armored copper power cable (*53*). (**B**) 3D view of the uncloaked configuration consisting solely of the superconducting (SC) layer, featuring sharp concave and convex edges and vertices. Magnetic field strength and contour lines are shown using a univocal color map (0 to 55 mT, blue to red). (**C**) 3D view of the optimized SC-SFM bilayer cloak using the spatially varying permeability profile $\mu_{r(SFM)}(\mathbf{r})$, achieving near-complete restoration of the background field. (**D**) Cross-sectional comparison of magnetic field lines and intensities before and after cloaking. The cloaking body and Cartesian coordinates are normalized and centered by the largest lobe of radius $R_1 = 31$mm, following the same design principles of the cylindrical cloak shown at Fig. 1. Relative dimensions can be found at the table S1. (**E**) Optimized SFM permeability profiles $\mu^{optimal}_{r(SFM)}(\mathbf{r})$ with ($\alpha = 0.3$) and without ($\alpha = 0$) regularization. The unregularized solution (left) exhibits large peaks (up to 166.4) and sharp spatial gradients across the domain, while the regularized profile (right) yields a smoother, less anisotropic distribution with substantially lower maxima. Notably, despite these differences, the normalized distortion metrics $\mathcal{E}^*$ indicate similarly effective cloaking performance in both cases.

difficult region to cloak (Fig. 3D). It is worth noting that in these complex designs, the original SC-only body may inherently create zero-field regions in space, acting as near-perfect magnetic shields, which limits the ability of any metastructure to fully restore the background field. This limitation is especially visible above the central lobe, where the positioning of the upper lobes contributes to these effects. Despite this, effective cloaking can still be achieved to a meaningful extent.

While perfect cloaking is more readily attainable in less convoluted shapes, such as those presented in Fig. 1, our framework remains capable of handling more geometrically complex cases, like the one shown in Fig. 3, through the design of optimized permeability profiles (Fig. 3E). In these cases, the normalized magnetic distortion metric remains below 10% ($\mathcal{E}^* < 10\%$), whether regularization is applied (right panel) or not (left panel). Applying regularization avoids the need for extremely anisotropic permeability values. For instance, without regularization, the required SFM permeability can reach $\mu_{r(SFM)} \simeq 166$, while with regularization a functional cloak can be realized with $\mu_{r(SFM)} \leq 49$, still achieving distortions below 1% at the edges of the region of interest Ω_{EXT}, i.e., at $x, y = \pm 2.5 \times R_1$. The most significant deviations occur only within a narrow zone, over the top two lobes of our anisotropic cloak design (~4% at $y = 2.5 \times R_1$), which is only caused by the conspicuous field shielding region formed in between. For a detailed breakdown of how the components of the background magnetic field are distorted by the SC layer and subsequently restored through the functional SC-SFM metastructure in all cases, the relative percentage magnetic deviation $B^*_{(x,y)} \in \Omega_{EXT}$ is shown in the Supplementary Materials at various longitudinal planes (Fig. S2).

DISCUSSION

We have developed a physics-driven optimization framework for designing magnetic cloaks based upon superconducting-ferromagnetic (SC-SFM) bilayers. Unlike existing approaches limited to idealized geometries, our method leverages full Maxwell equations to realize cloaking solutions for arbitrarily shaped structures. Validated against the cylindrical benchmark, the framework accurately reproduces known analytical results. It extends magnetic cloaking to noncircular and complex geometries—including faceted and multi-lobed designs—by optimizing spatially varying permeability profiles. A regularization scheme ensures that these solutions remain smooth and physically realizable, avoiding excessively high local permeability values and steep spatial gradients while maintaining low field

distortion ($\mathcal{E}^* < 10\%$). This flexibility paves the way for devising à la carte magnetic cloaks tailored to real-world components with complex shapes, such as triaxial power cables or shielded electronics. Because our simulations use parameters from commercially available superconductors, and the required magnetic permeabilities of the SFM layer remain within manufacturable ranges when regularization is applied, the experimental realization of functional SC-SFM cloaks of arbitrary shape is now within practical reach and will be presented in future work. Here, we recommend leveraging upon the knowledge acquired from established experimental setups in the study of cylindrical cloaks (*25*), where the inner superconducting shield could be constructed by fitting commercially available high-temperature superconducting (HTS) tapes to a custom 3D printed former of the desired geometry. The outer ferromagnetic shell could then be fabricated using a mouldable ferrite composite, such as a mixture of $Ni_{\delta}Zn_{1-\delta}Fe_2O_4$ powder and a nonmagnetic epoxy resin (*26*), allowing both the creation of arbitrary shapes and the tuning of the magnetic permeability of the SFM layer by adjusting the Ni/Zn ratio in the powder. Yet, we acknowledge several limitations that warrant future investigation. First, the practical fabrication of ferromagnetic materials with spatially varying and anisotropic permeability profiles remains a noteworthy engineering challenge. Second, the cloaking performance for nontrivial geometries may depend strongly on the direction of the incident magnetic field if the SC/SFM functional metastructure is not adequately tailored, i.e., a device optimized for one field orientation may require reoptimization to maintain its relative performance under different or rotating magnetic field directions, opening important future avenues of research. Last, it is worth mentioning that although the practical application of SC-SFM magnetic cloaks is bounded to the low temperatures of existing HTS materials, this should not undermine their viability as the cryogenics industry supporting superconducting technologies is already well established, with challenges that come more from the fundamental physics at low temperatures of the superconductors involved than the engineering challenges for achieving these temperatures (*27–30*). Thus, looking ahead, beyond the experimental validation of our current findings, the method we developed lays the foundation for next-generation 3D magnetic cloaks and raises the prospect of achieving cloaking using isotropic ferromagnetic materials through shape optimization alone. Such an approach could overcome many of the experimental hurdles associated with the fabrication of anisotropic SFM layers while also significantly reducing costs. Our framework also opens routes for investigating magnetic shielding in extreme field conditions, such as those found in fusion reactors or particle accelerators, where the protection of critical components is essential. In this light, our work not only generalizes the concept of magnetic cloaking through a physically and mathematically grounded model but also provides a computational toolset for its engineering realization.

MATERIALS AND METHODS

To model the magnetic behavior of the SC-SFM metastructure, we solve Maxwell's equations using a formulation similar—but not identical—to the classical H-formulation (*31*). In this approach, the magnetic field strength $\mathbf{H}$ is treated as the sole state variable, and the governing PDEs are solved accordingly. However, rather than using the conventional implementation found in platforms such as COMSOL Multiphysics, OPERA, or Quanscient, we adopt an optimal control framework inspired by methods developed for analyzing complex critical-state phenomena in type-II superconductors (*32*) due to the mathematical treatment required.

This approach inherently accounts for the behavior of practical superconductors, which do not exhibit ideal (fully diamagnetic) shielding but instead allow partial magnetic field penetration (*33*). The fundamental principles of the critical state theory underpinning our model have already been validated for cylindrical shell metastructures, where exact solutions to Maxwell's equations have shown that perfect magnetic cloaking remains achievable even at background fields, $|\mathbf{H}_{bg}|$, exceeding the full flux penetration threshold of the superconductor, H_{fp} (*34*, *35*). This occurs as long as $|\mathbf{H}_{fp}|$ is lower than the irreversibility field H_{irr}, i.e., below the magnetic field above which a type-II superconductor no longer shows irreversible magnetization due to vortex pinning (*32*). Therefore, although magnetic cloaking will still be possible for $|\mathbf{H}_{fp}| < |\mathbf{H}_{bg}| < |\mathbf{H}_{irr}|$, it is to be noticed that the object enclosed by the magnetic cloak will not be any longer shielded. Oppositely, for $|\mathbf{H}_{bg}| < |\mathbf{H}_{fp}|$, i.e., under partial flux penetration conditions, it has been already proven that the critical state theory effectively render to both magnetic cloaking and magnetic shielding (*33*).

Thus, assuming the magnetic permeability of the superconducting material is isotropic and equal to the vacuum permeability, $\mu_0 = 4\pi \times 10^{-7}$ H/m, the magnetic permeability of both SC and SFM regions can be expressed in terms of their relative permeability μ_r, neglecting any intrinsic magnetization. Under this assumption, the magnetic flux density becomes $\mathbf{B} = \mu_0\mu_r\mathbf{H}$, and Faraday's law reduces to

$$\nabla \times \mathbf{E} = -\mu_0\mu_r \frac{\partial \mathbf{H}}{\partial t} \tag{2}$$

Unlike the isotropic assumption for the SC, the relative permeability in the SFM domain, $\mu_{r(SFM)}$, is treated as a spatially varying function, independent of the magnetic field $\mathbf{H}$ (*36*), and is optimized to achieve magnetic cloaking. Using the magneto quasi-stationary (MQS) regime of the time-dependent Maxwell equations (*32*), where Ampère's law reduces to $\nabla \times \mathbf{H} = \mathbf{J}$, and applying Ohm's law $\mathbf{E} = \rho\mathbf{J}$ with $\mathbf{J}$ denoting current density, Eq. 2 can be rewritten solely in terms of the vector field $\mathbf{H}$ as

$$\mu_0\mu_r \frac{\partial \mathbf{H}}{\partial t} + \nabla \times (\rho \nabla \times \mathbf{H}) = 0 \tag{3}$$

In general, ferromagnets—similar to most conductors—obey Ohm's law with a linear relationship between electric field and current density. In contrast, superconductors exhibit a highly nonlinear relationship due to the distinct electronic nature of the current density, which remains a subject of ongoing research as it critically determines the applicability of many advanced superconductors (*37*). It is worth reminding that at least within the so-called quasi-steady low-frequency regime, i.e., for oscillating magnetic fields with frequencies below radio frequencies of up to 20 kHz, both the SC and the SFM can be modeled within the conceptual framework of the critical state theory that is to be followed below, with no relative differences in their electromagnetic performance (*36*, *38–40*). Thus, to represent the electrical properties of the SC, we adopt the widely used nonlinear $\mathbf{E} - \mathbf{J}$ power law, expressed as

$$\mathbf{E} = \rho\mathbf{J} = E_0\mathbf{J}\frac{|\mathbf{J}|^{n-1}}{J_c^{\,n}} \tag{4}$$

where E_0 is the electric field criterion used to define a unique critical current density J_c and n is an integer exponent that characterizes the steepness of the transition from the superconducting to the normal state. In our numerical experiments, we assume that the SC shell is composed of GdBCO sheets produced by SuNAM Co. Ltd., with experimentally measured parameters at *77* K: $J_c = 4.75 \times 10^{10}$ A/m^2, $n = 42$, and $E_0 = 1 \times 10^{-4}$ V/m (*31*, *41*).

Detailed geometric ratios for our modeled structures in Fig. 1 are summarized in table S1. For our computations, a uniform oscillating (ac) magnetic field of the form $H_{bg}(t) = H_a \sin(2\pi f \cdot t)$ is applied to the SC-SFM metastructure. The frequency is set to $f = 50$ Hz, corresponding to the standard electrical grid frequency in most regions of Europe, Africa, Asia, the United Kingdom, and Australia. The amplitude of the magnetic flux density, $B_a = \mu_0 H_a$, spans the range 10 mT $\leq B_a \leq$ 1.5 T, with the upper limit representative of the most widely used clinical magnetic resonance imaging systems worldwide, yet still below the irreversibility field reported for GdBCO thin films and coated conductors at *77* K, which lies in the range $B_{irr}^{c} \approx 2 - 10$ T (field along the c axis) and $B_{irr}^{ab} \approx 8 - 15$ T (field along the ab plane), increasing steeply as temperature decreases (*42*–*45*).

Furthermore, the superconducting layer thickness used in our models, $d \approx 0.26$ mm, matches the configuration experimentally validated by Gömöry *et al.* (*11*), where cylindrical magnetic cloaks were demonstrated. To provide additional context for the range of magnetic fields in which not only cloaking but also magnetic shielding can be achieved, it is worth recalling that for a cylindrical SC/SFM heterostructure with constant μ_r, a fully analytical solution for the full penetration field can be derived. In particular, it has been shown (*34*, *35*) that if the inner radius of the SC shell is R_0, the full penetration field is given by

$$B_{fp} = \frac{2\mu_0}{\pi} J_c (R_1 - R_0) \left\{ 1 + \left(\frac{R_2^2 - R_1^2}{R_2^2} \right) \left[\frac{(\mu_r - 1)(2\mu_r - 1)}{6\mu_r} + \frac{(\mu_r^2 - 1)}{12\mu_r} \frac{R_0 (R_0 + R_1)}{R_1^2} \right] \right\} \quad (5)$$

In our specific case for the cylindrical cloak, this yields $B_{fp} \approx 12.98$ T, indicating that all magnetic fields B_{bg} used in our simulations lie well below this threshold. This confirms that the system operates in the partial penetration regime, enabling both magnetic cloaking and shielding within the cavity of the metastructure.

Moreover, since in all our cases $B_{fp} \gg B_a$, and considering that the Eq. 4 for the SuNAM tapes makes the $\mathbf{E} - \mathbf{J}$ power law nearly indistinguishable from the critical state theory (*37*), the resulting area of the SC with magnetization currents (i.e., penetrated field) remains extremely narrow, effectively behaving as if the SC was in the Meissner state. Then, reminding that under these conditions, the cloaking performance becomes independent of the applied magnetic field intensity (*10*); this explains why, despite the use of a time-dependent oscillating field, our simulations show negligible differences in cloaking performance even for geometries of different shapes but comparable relative dimensions to the cylindrical benchmark (see also figs. S2 and S3). Last, it is worth emphasizing that the flexibility of our model to accommodate arbitrary geometries and materials allows our optimization framework to be used in conjunction with the analytical expression in Eq. 5 as a practical design benchmark. Specifically, it enables users to scope initial design dimensions for the SC and SFM layers to ensure effective magnetic cloaking over the range of applied field intensities relevant to their intended applications. However, we note that for $B_a > B_{fp}$, magnetic shielding inside the cloaked region may become incomplete, even if the external field distortions remain well minimized, that is, cloaked.

Thus far, the physical principles and mathematical framework used remain conventional and are consistent with previous applications to SC-SFM metastructures (*36*, *38*, *46*). However, a notable distinction in the present formulation is that the magnetic permeability of the SFM material, $\mu_{r(SFM)}$, is regarded as a nonisotropic spatial variable, which must be optimized to achieve cloaking performance, thereby necessitating a dedicated optimization step.

Let $\Omega \subseteq \mathbb{R}^3$ denote the computational domain encompassing the SC, SFM, and surrounding medium where the magnetic field $\mathbf{H}$ is computed. We assume Ω to be a Lipschitz domain, that is, a region whose boundary is "sufficiently regular," locally representable as the graph of a Lipschitz-continuous function. To express Maxwell's equations in weak form, we define the Hilbert space

$$H(\text{curl};\Omega) := \left\{ \mathbf{u} \in \left[L^2(\Omega)\right]^3 : \nabla \times \mathbf{u} \in \left[L^2(\Omega)\right]^3 \right\} \quad (6)$$

along with its subspace

$$H_0(\text{curl};\Omega) := \{ \mathbf{u} \in H(\text{curl};\Omega) : \mathbf{u} \times \mathbf{n}|_{\partial\Omega} = 0 \} \quad (7)$$

which consists of fields with vanishing tangential components on the boundary $\partial\Omega$.

With these definitions in place, the weak formulation of the H-based representation of Faraday's law in Eq. 2 is given by

$$\text{Find } \mathbf{H} \in H(curl, \Omega) \text{ such that } \mathbf{H}(t) \times \mathbf{n} = \mathbf{g}(t) \text{ on } \partial\Omega, \text{ and } F(\mathbf{H}) := \int_{\Omega} \mu_0 \mu_r \frac{\partial \mathbf{H}}{\partial t} \cdot \mathbf{W} + \rho \; (\nabla \times \mathbf{H}) \cdot (\nabla \times \mathbf{W}) \; dr = 0 \text{ for all } \mathbf{W} \in H_0(curl, \Omega) \quad (8)$$

where $gH_{bg} \times \mathbf{n}$ and $\mathbf{H}_{bg}$ denote a time-dependent background magnetic field, i.e., the uniform applied field to the functional metastructure $\mathbf{H}_{bg} = \mu_0^{-1} B_a \sin(\omega t)\hat{j}$. Consequently, the properties of the function spaces defined in Eqs. 6 and 7 ensure the continuity of both $\mathbf{H} \times \mathbf{n}$ and $\rho \; (\nabla \times \mathbf{H}) \times \mathbf{n}$ across all interfaces, satisfying the physical boundary conditions of Ampère's law while solving Faraday's law in the form of Eq. 8.

Since μ_r is a space-dependent variable, we introduce an objective functional $\mathcal{E}\left[\mathbf{H}, \mu_{r(SFM)}\right]$ to quantify how effectively a candidate permeability distribution $\mu_{r(SFM)}(\mathbf{r})$ achieves magnetic cloaking. This cloaking functional measures the distortion of the magnetic field in a predefined region outside the SC-SFM bilayer

$$\mathcal{E}\left[\mathbf{H}, \mu_{r(SFM)}\right] \int_0^T \int_{\Omega_{EXT}} \left\| \mathbf{H}\left[\mu_{r(SFM)}\right] - \mathbf{H}_{bg} \right\|^2 \; dr \; dt \quad (9)$$

where $\|\cdot\|$ denotes the Euclidean norm, $\mathbf{H}\left[\mu_{r(SFM)}\right]$ is the solution of Eq. 8 corresponding to a given permeability distribution, and Ω_{EXT} is a region of interest surrounding the metastructure. In simple terms, the closer $\mathbf{H}\left[\mu_{r(SFM)}\right]$ is to the undisturbed background field $\mathbf{H}_{bg}$, the more effective the cloaking. Perfect cloaking corresponds to $\mathcal{E}\left[\mathbf{H}, \mu_{r(SFM)}\right] = 0$, and minimizing $\mathcal{E}$ provides a quantitative means to optimize $\mu_{r(SFM)}$ over the time interval $[0, T]$.

To identify an optimal permeability $\mu_{r(SFM)}$ for cloaking, we minimize Eq. 9 subject to the PDE constraint (*8*), i.e., we solve the PDE-constrained optimization problem

$$\min_{\mu_{r(SFM)} \in L^2(\Omega_{SFM})} \mathcal{E}\left[\mathbf{H}, \mu_{r(SFM)}\right] + \alpha \ \mathcal{R}_\epsilon\left[\mu_{r(SFM)}\right] \text{ subject to } F\left[\mathbf{H}, \mu_{r(SFM)}\right] = 0 \tag{10}$$

where Ω_{SFM} denotes the region occupied by the SFM shell, $\alpha \geq 0$ is a regularization parameter, and $\mathcal{R}_\epsilon\left[\mu_{r(SFM)}\right]$ is a regularization term penalizing excessive spatial variation in $\mu_{r(SFM)}$. Specifically, we adopt a smoothed total variation functional

$$\mathcal{R}_\epsilon\left[\mu_{r(SFM)}\right] := \int_{\Omega_{SFM}} \sqrt{\epsilon + \|\nabla \mu_{r(SFM)}(\mathbf{r})\|^2} \ \mathrm{d}r \tag{11}$$

where $\epsilon > 0$ is a smoothing parameter introduced to ensure differentiability. The optimization problem in Eq. 10 is solved using the finite element software Firedrake (*47*), the automated differentiation library Pyadjoint (*48*), and the BFGS algorithm from SciPy (*49*), with $\epsilon = 0.01$ as recommended. The PDE constraint is discretized using quadratic Nédélec elements of the second kind (*50*), and the resulting nonlinear problem is solved via PETSc's implementation of Newton's method (*51*).

Supplementary Materials

This PDF file includes:

Figs. S1 to S3

Table S1

REFERENCES

1. J. B. Pendry, D. Schurig, D. R. Smith, Controlling electromagnetic fields. *Science* **312**, 1780–1782 (2006).
2. U. Leonhardt, Optical conformal mapping. *Science* **312**, 1777–1780 (2006).
3. A. Alù, N. Engheta, Achieving transparency with plasmonic and metamaterial coatings. *Phys. Rev. E Stat. Nonlinear Soft Matter Phys.* **72**, 016623 (2005).
4. S. A. Cummer, B. I. Popa, D. Schurig, D. R. Smith, J. Pendry, M. Rahm, A. Starr, Scattering theory derivation of a 3D acoustic cloaking shell. *Phys. Rev. Lett.* **100**, 024301 (2008).
5. T. Chen, C. N. Weng, J. S. Chen, Cloak for curvilinearly anisotropic media in conduction. *Appl. Phys. Lett.* **93**, 114103 (2008).
6. F. Yang, Z. L. Mei, T. Y. Jin, T. J. Cui, Dc electric invisibility cloak. *Phys. Rev. Lett.* **109**, 053902 (2012).
7. B. Wood, J. B. Pendry, Metamaterials at zero frequency. *J. Phys. Condens. Matter* **19**, 076208 (2007).
8. G. Alekseev, A. Tokhtina, O. Soboleva, Invisibility problem in acoustics, electromagnetism and heat transfer. Inverse design method. *J. Phys. Conf. Ser.* **894**, 012004 (2017).
9. S. Xu, Y. Wang, B.-l. Zhang, H. Chen, Invisibility cloaks from forward design to inverse design. *Sci. China Inf. Sci.* **56**, 1–11 (2013).
10. A. Sanchez, C. Navau, J. Prat-Camps, D.-X. Chen, Antimagnets: Controlling magnetic fields with superconductor-metamaterial hybrids. *New J. Phys.* **13**, 093034 (2011).
11. F. Gömöry, M. Solovyov, J. Šouc, C. Navau, J. Prat-Camps, A. Sanchez, Experimental realization of a magnetic cloak. *Science* **335**, 1466–1468 (2012).
12. J. Šouc, M. Solovyov, F. Gömöry, J. Prat-Camps, C. Navau, A. Sanchez, A quasistatic magnetic cloak. *New J. Phys.* **15**, 053019 (2013).
13. M. Solovyov, J. Šouc, F. Gömöry, Magnetic cloak for low frequency AC magnetic field. *IEEE Trans. Appl. Supercond.* **25**, 1–5 (2014).
14. J. Zhu, W. Jiang, Y. Liu, G. Yin, J. Yuan, S. He, Y. Ma, Three-dimensional magnetic cloak working from d.c. to 250 kHz. *Nat. Commun.* **6**, 8931 (2015).
15. N. Rotheudt, M. Fracasso, P. Vanderbemden, L. Gozzelino, Superconductor-based passive shielding and screening systems. *Supercond. Sci. Technol.* **38**, 043002 (2025).
16. J. Zhan, K. Li, Y. Zhou, X. Liu, Y. Ma, Ultrathin conformal magnetic invisible cloak for irregular objects. *ACS Appl. Mater. Interfaces* **13**, 17104–17109 (2021).
17. J. Zhan, Y. Mei, K. Li, Y. Zhou, J. Chen, Y. Ma, Conformal metamaterial coats for underwater magnetic-acoustic bi-invisibility. *Appl. Phys. Lett.* **120**, 094104 (2022).
18. G. V. Alekseev, Control of boundary impedance in two-dimensional material-body cloaking by the wave flow method. *Comput. Math. Math. Phys.* **53**, 1853–1869 (2013).
19. G. V. Alekseev, Cloaking via impedance boundary condition for the 2-D Helmholtz equation. *Appl. Anal.* **93**, 254–268 (2014).
20. G. V. Alekseev, Stability estimates in the problem of cloaking material bodies for Maxwell's equations. *Comput. Math. Math. Phys.* **54**, 1788–1803 (2014).
21. G. V. Alekseev, Analysis and optimization in problems of cloaking of material bodies for the Maxwell equations. *Differ. Equ.* **52**, 361–372 (2016).
22. G. V. Alekseev, Y. E. Spivak, Theoretical analysis of the magnetic cloaking problem based on an optimization method. *Differ. Equ.* **54**, 1125–1136 (2018).
23. G. V. Alekseev, Y. E. Spivak, A. V. Lobanov, I. V. Vinogradov, Optimization-based analysis of inverse problems of designing magnetic cloaking and shielding devices, in *2019 International Conference on Electromagnetics in Advanced Applications (ICEAA)* (IEEE, 2019), pp. 0095–0098.
24. G. V. Alekseev, Y. E. Spivak, Optimization-based numerical analysis of three-dimensional magnetic cloaking problems. *Comput. Math. Math. Phys.* **61**, 212–225 (2021).
25. M. Solovyov, J. Souc, J. Kovac, F. Gomory, E. Mikulasova, M. Usakova, E. Usak, Design of magnetic cloak for experiments in AC regime. *IEEE Trans. Appl. Supercond.* **26**, 1–6 (2016).
26. N. Idayanti, Dedi, T. Kristiantoro, N. Sudrajat, D. Mulyadi, A. Gustinova, Effects of varying chemical composition with x = 0.1 - 0.7 on magnetic properties of soft ferrite $Ni_{1\text{-}x}Zn_xFe_2O_4$. *IOP Conf. Ser. Mater. Sci. Eng.* **620**, 012105 (2019).
27. T. A. Coombs, Q. Wang, A. Shah, J. Hu, L. Hao, I. Patel, H. Wei, Y. Wu, T. Coombs, W. Wang, High-temperature superconductors and their large-scale applications. *Nat. Rev. Electr. Eng.* **1**, 788–801 (2024).
28. E. W. Stautner, K. S. Haran, P. J. Ansell, C. Minas, *Aircraft Cryogenics* (Springer Nature, 2024).
29. S. Alam, M. S. Hossain, S. R. Srinivasa, A. Aziz, Cryogenic memory technologies. *Nat. Electr.* **6**, 185–198 (2023).
30. H. Chang, J. Zhang, Refrigeration technologies of cryogenic chips. *Chip* **2**, 100054 (2023).
31. M. Clegg, H. N. H. Al-Ssalih, H. S. Ruiz, Magnetization losses of transposed stacked tape conductors under AC transverse magnetic fields. *IEEE Access* **13**, 84013–84023 (2025).
32. A. Badía-Majós, C. López, H. S. Ruiz, General critical states in type-II superconductors. *Phys. Rev. B* **80**, 144509 (2009).
33. J. Peña-Roche, Y. A. Genenko, A. Badía-Majós, Magnetic invisibility of the magnetically coated Type-II superconductor in partially penetrated state. *Appl. Phys. Lett.* **109**, 092601 (2016).
34. S. V. Yampolskii, Y. A. Genenko, Magnetic detectability of a finite size paramagnet/superconductor cylindrical cloak. *Appl. Phys. Lett.* **104**, 033501 (2014).
35. S. V. Yampolskii, Y. A. Genenko, Magnetic cloaking by a paramagnet/superconductor cylindrical tube in the critical state. *Appl. Phys. Lett.* **104**, 143504 (2014).
36. M. U. Fareed, H. S. Ruiz, Critical state theory for the magnetic coupling between soft ferromagnetic materials and type-II superconductors. *Materials* **14**, 6204 (2021).
37. H. Ruiz, J. Hanisch, M. Polichetti, A. Galluzzi, Critical current density in advanced superconductors. *Prog. Mater. Sci.* **155**, 101492 (2025).
38. M. Kapolka, H. S. Ruiz, Maximum reduction of energy losses in multicore MgB_2 wires by metastructured soft-ferromagnetic coatings. *Sci. Rep.* **12**, 7030 (2022).
39. M. W. Coffey, High-frequency linear response of anisotropic type-II superconductors in the mixed state. *Phys. Rev. B* **47**, 12284–12287 (1993).
40. R. Prozorov, A. Shaulov, Y. Wolfus, Y. Yeshurun, Frequency dependence of the local ac magnetic response in type-II superconductors. *Phys. Rev. B* **52**, 12541–12544 (1995).
41. K. Osamura, Mechanism for the uniaxial strain dependence of the critical current in practical REBCO tapes. *Supercond. Sci. Technol.* **29**, 065019 (2016).
42. K. J. Song, J. S. You, S. K. Lee, J. H. Lee, H. Lee, S. H. Moon, The superconducting properties of several HTS GdBCO coated conductors. *IEEE Trans. Appl. Supercond.* **35**, 1–6 (2025).
43. P. Cayado, M. Erbe, S. Kauffmann-Weiss, A. Jung, J. Hänisch, B. Holzapfel, Chemical solution deposition of $Y_{1\text{-}x}Gd_xBa_2Cu_3O_{7\text{-}\delta}$-$BaHfO_3$ nanocomposite films: Combined influence of nanoparticles and rare-earth mixing on growth conditions and transport properties. *RSC Adv.* **8**, 42398–42404 (2018).
44. M. Miura, B. Maiorov, F. F. Balakirev, T. Kato, M. Sato, Y. Takagi, T. Izumi, L. Civale, Upward shift of the vortex solid phase in high-temperature-superconducting wires through high density nanoparticle addition. *Sci. Rep.* **6**, 20436 (2016).
45. S. Rosenzweig, J. Hänisch, K. Iida, A. Kauffmann, C. Mickel, T. Thersleff, J. Freudenberger, R. Hühne, B. Holzapfel, L. Schultz, Irreversibility field up to 42 T of $GdBa_2Cu_3O_{7\text{-}\delta}$ thin films grown by PLD and its dependence on deposition parameters. *Supercond. Sci. Technol.* **23**, 105017 (2010).
46. M. U. Fareed, B. C. Robert, H. S. Ruiz, Electric field and energy losses of rounded superconducting/ferromagnetic heterostructures at self-field conditions. *IEEE Trans. Appl. Supercond.* **29**, 1–5 (2019).
47. D. A. Ham, P. H. J. Kelly, M. Lawrence, C. Cotter, R. C. Kirby, *Firedrake User Manual* (Imperial College London, University of Oxford, Baylor University, and University of Washington, ed. 1, 2023).
48. S. K. Mitusch, S. W. Funke, J. S. Dokken, dolfin-adjoint 2018.1: Automated adjoints for FEniCS and Firedrake. *J. Open Source Softw.* **4**, 1292 (2019).
49. P. Virtanen, R. Gommers, T. E. Oliphant, M. Haberland, T. Reddy, D. Cournapeau, E. Burovski, P. Peterson, W. Weckesser, J. Bright, S. J. van der Walt, M. Brett, J. Wilson,

K. J. Millman, N. Mayorov, A. R. J. Nelson, E. Jones, R. Kern, E. Larson, C. J. Carey, İ. Polat, Y. Feng, E. W. Moore, J. V. Plas, SciPy 1.0 Contributors, SciPy 1.0: Fundamental algorithms for scientific computing in Python. *Nat. Methods* **17**, 261–272 (2020).
50. J.-C. Nédélec, A new family of mixed finite elements in $\mathbb{R}^3$. *Numer. Math.* **50**, 57–81 (1986).
51. S. Balay, S. Abhyankar, M. Adams, J. Brown, P. Brune, K. Buschelman, L. Dalcin, A. Dener, V. Eijkhout, W. Gropp, D. Karpeyev, D. Kaushik, M. Knepley, D. May, L. Curfman Mc Innes, R. Mills, T. Munson, K. Rupp, P. Sanan, B. Smith, S. Zampini, H. Zhang, H. Zhang, PETSc (2015); https://publications.anl.gov/anlpubs/2019/12/155920.pdf.
52. C. Lee, H. Son, Y. Won, Y. Kim, C. Ryu, M. Park, M. Iwakuma, Progress of the first commercial project of high-temperature superconducting cables by KEPCO in Korea. *Supercond. Sci. Technol.* **33**, 044006 (2020).
53. X. Xu, J. Ding, Q. Ding, Q. Wang, Y. Xun, A study on the detection of conductor quantity in cable cores based on YOLO-cable. *Sci. Rep.* **14**, 31107 (2024).

Acknowledgments: H.S.R. thanks F. Gömöry and M. Soloviov for engaging discussions and suggestions regarding the manufacturability of the magnetic cloaks and A. Badía-Majós for further discussions on the limits and scope of existent analytical solutions. **Funding:** This work was supported by the The UK Research and Innovation, Engineering and Physical Sciences Research Council (EPSRC) grant reference EP/S025707/1 (to H.S.R.); the European Cooperation in Science and Technology (COST) action CA19108 (to Y.G. and H.S.R.); and the University of Leicester, Doctoral College, Future 100 Studentship (to Y.G.). **Author contributions:** Conceptualization: H.S.R., and A.P. Methodology: H.S.R. and A.P. Investigation: H.S.R., A.P., and Y.G. Visualization: H.S.R. and Y.G. Funding acquisition: H.S.R. and A.P. Project administration: H.S.R. and A.P. Supervision: H.S.R. and A.P. Writing—original draft: A.P. and Y.G. Writing—review and editing: H.S.R. **Competing interests:** The authors declare that they have no competing interests. **Data and materials availability:** All data and code needed to evaluate and reproduce the results in the paper are present in the paper and/or the Supplementary Materials. Source data are available in doi: 10.5281/zenodo.17250275.

Submitted 1 July 2025
Accepted 18 November 2025
Published 19 December 2025
10.1126/sciadv.aea2468
