# Supplementary material for: Designing functional magnetic cloaks for real-world geometries
Source: Sci Adv. 2025 Dec 19;11(51):eaea2468. doi: 10.1126/sciadv.aea2468 (PMC12716416; doi:10.1126/sciadv.aea2468)
Supplement: Supplementary file 2 — Figs. S1 to S3 Table S1 [file sciadv.aea2468_sm.pdf]

Science Advances
AAAS

Supplementary Materials for

Designing functional magnetic cloaks for real-world geometries

Yusen Guo *et al.*

Corresponding author: Alberto Paganini, a.paganini@leicester.ac.uk; Harold S. Ruiz, dr.harold.ruiz@leicester.ac.uk

Sci. Adv. **11**, eaea2468 (2025)
DOI: 10.1126/sciadv.aea2468

This PDF file includes:

Figs. S1 to S3
Table S1

Supplementary Materials

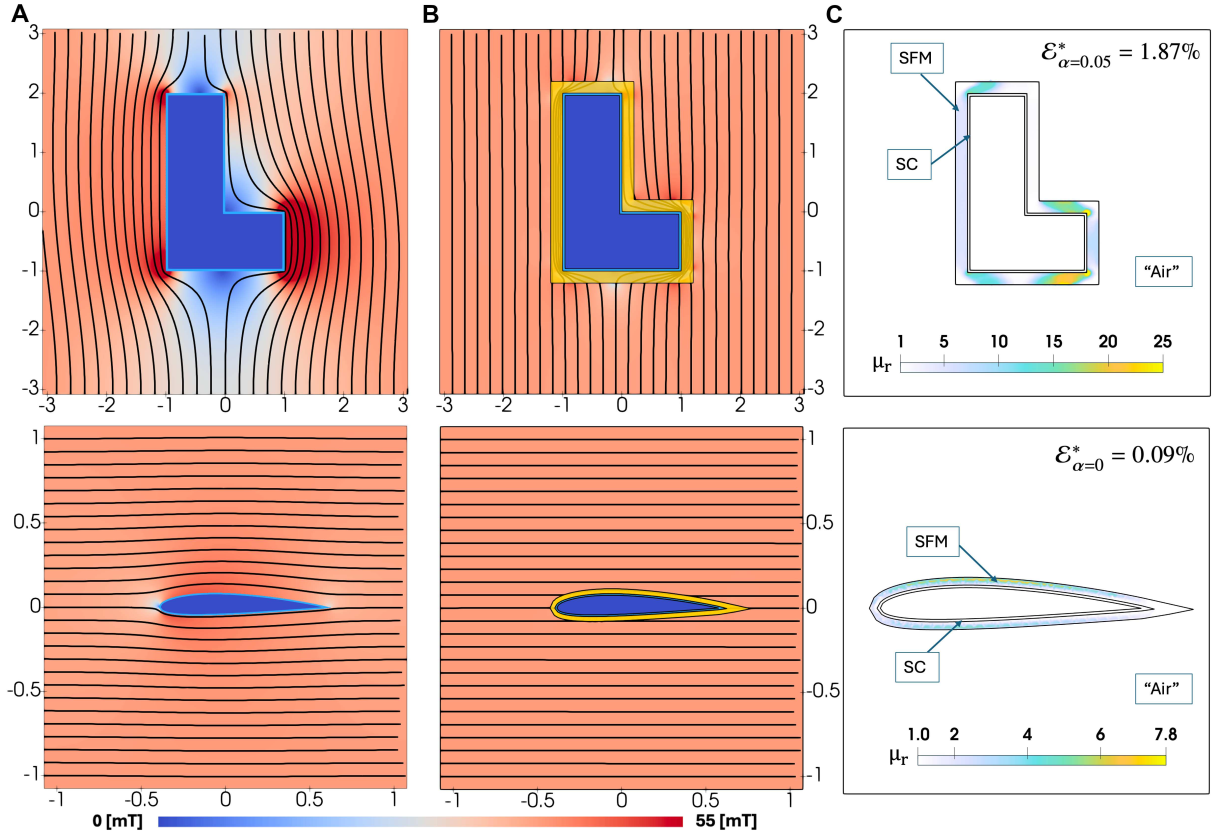

Figure S1: Magnetic cloaking in further bilayer SC-SFM metastructures with non-symmetric geometries. (**A**) Magnetic field response by a superconducting sheet of finite thickness enclosing (top) an L-shaped cross-section and (bottom) a NACA-2414 cambered aerofoil profile, both exhibiting pronounced shielding/diamagnetic effects seen as distortions in the orientation and intensity of the originally constant background magnetic field. (**B**) Corresponding cloaked configurations using optimized SC-SFM bilayers. The light blue inner shell represents the superconducting (SC) layer, while the yellow outer shell denotes the soft-ferromagnetic (SFM) layer. The applied magnetic field is oriented vertically for the L-shape and horizontally for the aerofoil, each with amplitude B_a = 40 mT and frequency 50 Hz. The functional SFM layer counteracts the distortions introduced by the SC layer alone, restoring the uniform background field. (**C**) Spatially optimized permeability distributions $\mu_{r,\mathrm{opt}}(\boldsymbol{x})$ for each configuration. For the L-shaped cloak, regularization ($\alpha = 0.05$) in Eq. 10 was applied to limit the high permeability values required at corners, keeping most values below 25. For the aerofoil geometry, no regularization was needed, yielding a smooth distribution ranging from 1 to 7.8. The resulting distortion metrics confirm effective cloaking: $\mathcal{E}^* = 1.87\%$ for the L-shape and $\mathcal{E}^* = 0.09\%$ for the aerofoil.

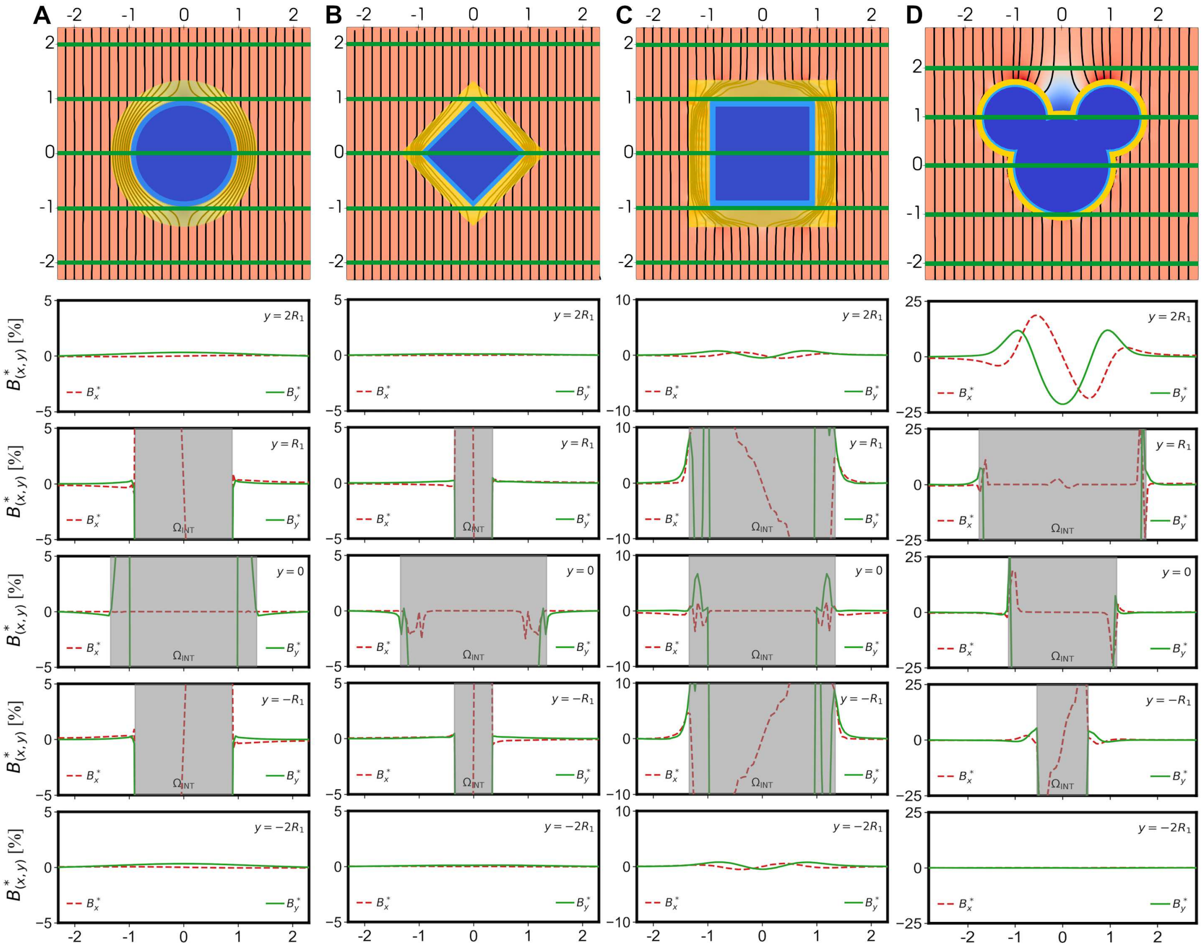

Figure S2: Relative effectiveness of magnetic cloaking based on the normalized magnetic flux deviation. The relative deviation of the magnetic field from the applied background is quantified as $B^*_{(x,y)} = \left|B_{(x,y)} - B_{\mathrm{bg},(x,y)}\right| / \left|\mathbf{B}_{bg}\right| \times 100\%$, evaluated at selected cross-sectional planes for SC–SFM cloaks optimized for pipe geometries with (**A**) cylindrical, (**B**) diamond, (**C**) square, and (**D**) strongly anisotropic cross-sections. In all simulations, a uniform background field is applied along the y-axis, such that $\mathbf{B}_{\mathrm{bg}} = B_a \sin(\omega t)\, \hat{\jmath}$ throughout the domain. At the peak of the sinusoidal field (i.e., $\mathbf{B}_{\mathrm{bg}} = B_a\, \hat{\jmath}$), the local relative deviations in the x- and y-components of the magnetic flux density are computed as: $B^*_x = B_{x,\in\Omega_{\mathrm{EXT}}}/B_a \times 100\%$ and $B^*_y = (B_y - B_a)_{\in\Omega_{\mathrm{EXT}}}/B_a \times 100\%$. The top row displays the geometries and corresponding field lines as shown in Figs. 1 & 3 of the main manuscript, with added green reference planes indicating the five y-levels at which deviations are measured. From top to bottom, rows two through six show B^*_x (dashed lines) and B^*_y (solid lines) along the x-axis at $y = 2R_1$, R_1, 0, $-R_1$, and $-2R_1$, respectively. The shadowed regions (Ω_{INT}) represent internal areas to the functional metastructure where the effects of the SC and SFM shells can be seen, while cloaking region of interest refer to any region outside of the metastructure ($\Omega_{\mathrm{EXT}} \notin \Omega_{\mathrm{INT}}$). Displayed results correspond to the unregulated $\mathcal{E}^*_{\alpha=0}$ solutions. Perfect magnetic cloaking is achieved when both B^*_x and B^*_y become negligible across Ω_{EXT}, as demonstrated in the cylindrical configuration, which also admits an analytical solution.

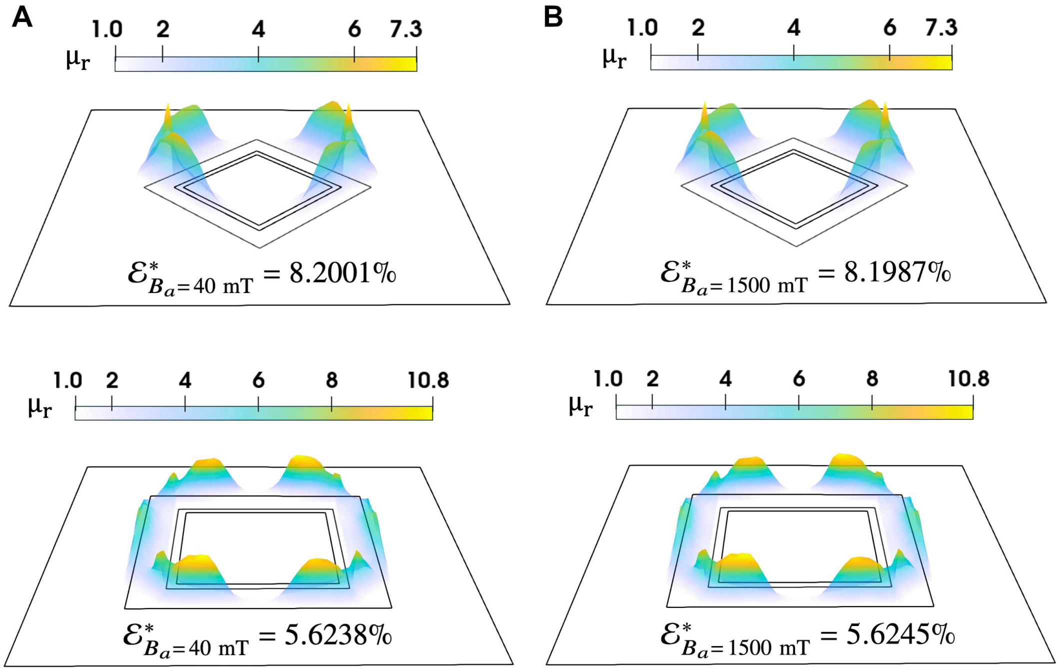

Figure S3: Optimized permeability profiles for functional magnetic cloaks under different field intensities. Top row: diamond-shaped cloak; **bottom row**: square-shaped cloak. **Left column** (**A**): 3D visualizations of the optimized permeability distributions $\mu_{r(\mathrm{SFM})}$ obtained with regularization ($\alpha = 0.5$ for the diamond, $\alpha = 0.25$ for the square) under an applied sinusoidal magnetic field of amplitude $B_a = 40$ mT and frequency 50 Hz. **Right column** (**B**): The same but, with the optimized permeability profiles computed under a substantially higher applied field of $B_a = 1.5$ T at the same frequency. The resulting distributions are virtually identical across both field strengths, with the normalized distortion metric differing by less than 0.001%. This confirms that the developed optimization framework may consistently converge to the same optimal permeability profiles across a wide range of magnetic field amplitudes and geometries.

Table S1: Relative dimensions of the devised SC-SFM functional cloaks.

**Cloaking Body**	R_0 [mm]†	R_1 [mm]‡	R_2 [mm]§
Cylindrical	6.25	6.51	8.72
Square / Diamond *	6.25	6.51	8.72
Multi-lobed (Lateral lobes)	20	21	25
Multi-lobed (Central lobe)	30	31	35

† R_0 generally defines the inner radius (or respective length*) of the hollowed SC layer of the devised cloaking geometry.

‡ R_1 generally defines the outer radius (or respective length*) of the hollowed SC layer of the devised cloaking geometry, being then $R_1 - R_0$ the assumed thickness of the SC shell. This also defines the inner radius of the hollowed SFM domain.

§ R_2 generally defines the outer radius (or respective length*) of the hollowed SFM layer of the devised cloaking geometry, being then $R_2 - R_1$ the assumed thickness of the SFM shell.

* Geometrical dimensions for the square ($\square$) and diamond ($\diamond$) shapes which produce rather different scenarios for SC shielding, are both based on the relative definition of R_0 being it: half of the side-length for the square geometry and, half of the height or width for the diamond geometry, such that $R_0(\square) = R_0(\diamond)$.
